# Supplementary material for: Association between oral microbiome and seven types of cancers in East Asian population: a two-sample Mendelian randomization analysis
Source: Front Mol Biosci. 2023 Nov 21;10:1327893. doi: 10.3389/fmolb.2023.1327893 (PMC10702768; doi:10.3389/fmolb.2023.1327893)
Supplement: Supplementary file 1 [file DataSheet1.ZIP › Supplementary Materials/MR plots of saliva/saliva═╝/Pancreatic cancer/pheno.2204_to_pancreatic cancer_leave_one_out.pdf]

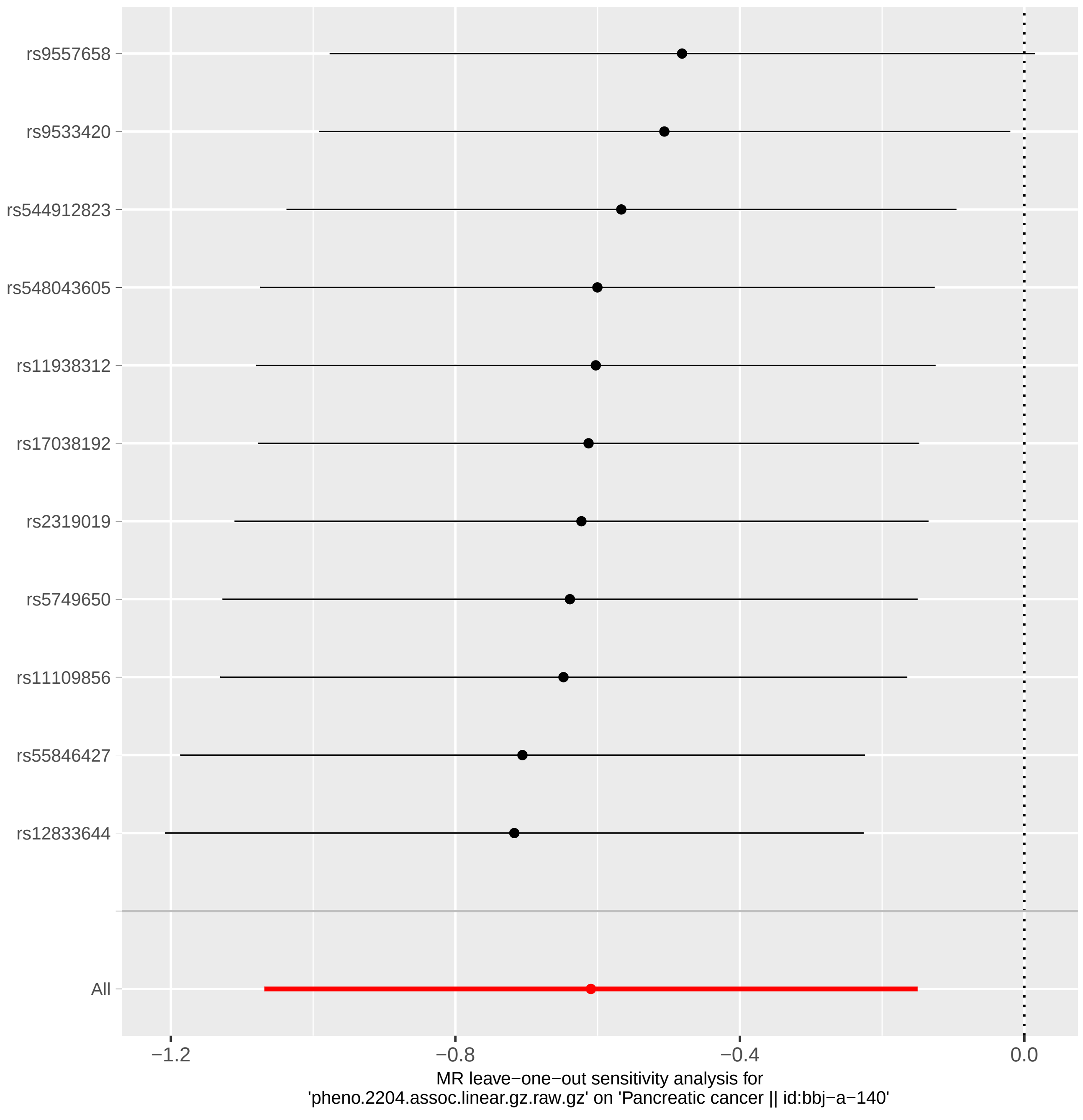

rs9557658
rs9533420
rs544912823
rs548043605
rs11938312
rs17038192
rs2319019
rs5749650
rs11109856
rs55846427
rs12833644
All
−1.2
−0.8
−0.4
0.0
MR leave−one−out sensitivity analysis for
'pheno.2204.assoc.linear.gz.raw.gz' on 'Pancreatic cancer || id:bbj−a−140'
